# Statement by Dr. Addison

**Published:** 1918-10-05

**Authors:** 


### THE RECONSTRUCTION OF THE PROFESSIONS.

### Statement by Dr. Addison.

The inaugural address at the opening meeting of the seventy-seventh session of the School of Pharmacy of the Pharmaceutical Society, on Wednesday, October 2, was delivered by Dr. Addison, Minister of Reconstruction, and in the course of his speech he made an important statement with regard to the deferred training of students, not only in pharmacy, but in other professions, whose studies had been interrupted by the call to active service.

### A Modified Curriculum.

Addressing the students, among whom was a large proportion of women, Dr. Addison said there would be many young men who, but for their absence fighting their battles, would by now have passed their qualifying examination. It was the duty of the State, just as it was the duty of the Pharmaceutical Society, to minimise as much as possible the effect of these months and years of absence from their colleges, dispensaries, pharmacies, and laboratories. That handicap, he said, must be reduced, not only because sheer justice to the men demanded it, but because the community would require their services, and must see to it that they were equipped for service. Anything like "stickiness" on the part of the professions, continued Dr. Addison, in revising temporarily the various requirements from candidates for full qualification—subject, of course, to satisfactory fitness being shown—would be the most foolish of policies. Looked at from the lowest standpoint, such an attitude would only result in laying up trouble for the future for any profession which adopted it. Endless difficulties would ensue if upon the flanks of the recognised professions a body of men should be created partly trained in the work of the particular profession but without the sanction of a completed course. He added that *the Government had now under consideration detailed proposals for dealing with these matters.*

### Its Shortening for Practitioners and Chemists.

**The importance of this official statement is obvious. It would seem to imply that the Government is favourably disposed towards the policy of modifying the existing regulations regarding the duration of study prior to qualification. Briefly, we take it that Dr. Addison means that in order to bring the ratio of doctors and chemists to the population up to the normal, plans for shortening the curriculum are under consideration. But until these plans are disclosed by the Government it will not be of much value to say anything by way of comment.**

### Women Pharmacists.

Proceeding with his address, Dr. Addison said that, as in all other professions, so in pharmacy, the war had brought about a serious break in the steady flow of yearly entrants, but in pharmacy, as in many other callings, war-time had seen women undertaking a greater share of the work. He found that in the years 1917 and 1918 44 per cent. of those who had qualified as pharmacists were women, and the honours lists exhibited striking testimony to the capacity of women students. Women had begun to enter their ranks in pre-war days, but during the war the relative proportion of women to men entering the School of Pharmacy had been more than reversed. How far this condition might be transitory time would prove. But the war had brought to women conceptions of capacity for service, with its corresponding responsibility, and a sense of power which would make it impossible in future to shut them out of spheres of occupations for which they had proved their fitness.

### Capturing the German Drug Trade.

From the welcome invasion by women of the pharmaceutical calling Dr. Addison passed on to the important part played by pharmacy in the production of drugs which had formerly been a monopoly of the Germans. He spoke of the rude awakening that this country had soon after we went to war because of our depending on Germany for many of the most commonly prescribed drugs and chemicals, and of the methods which had been successfully taken to cope with the difficulties arising from this cause. The result, he said, was that today there were none of those medicinal preparations of great importance that were not being made in amply sufficient quantities in this country. As examples of these he mentioned aspirin, salicylic acid, and novocain. Much progress had also been made in the home production of many wild plants, such as belladonna, so that the supply of many of these is fully adequate to our needs. The story of their combat with the deficient drug supply would provide a most interesting and attractive example of many of their national achievements during the war. The particular lesson standing out therefrom was that they must have a much better supply of trained chemists. But when they found, as they did at the beginning of the war, the British Government advertising for trained chemists at a salary of £3 per week, they had a striking example of the kind of estimate which the country at that time had formed as to the place of science in industry. In that respect, as, of course, in many others, enterprising and enlightened firms were often far in advance of the Government, and he was disposed to think that industry in future would offer wide and well-paid opportunities to highly-trained chemists. It would be bad for their industrial outlook if it did not. But science in industry was not to be bottled up in laboratories. It needed a wider range and a freer field, so as to be able to survey in full detail methods of production and manufacturing processes in all their ramifications.

